# A Comparison of the Psychometric Properties of PROMIS Computer Adaptive Tests and Short Forms Vs Legacy Patient-Reported Outcome Measures in Total Knee Arthroplasty Patients

**DOI:** 10.1016/j.artd.2026.101964

**Published:** 2026-03-06

**Authors:** Christel Braaksma, Nienke Wolterbeek, Maurits Remmelt Veen, Rudolf Wilhelm Poolman, Yvette Pronk, Ariena Jorien Rasker, Raymond Willem Jozef Gerardus Ostelo, Caroline Barbara Terwee

**Affiliations:** a Department of Orthopedic Surgery, St. Antonius Hospital, Utrecht, The Netherlands; b Joint Research Department, OLVG, Amsterdam, The Netherlands; c Department of Orthopedic Surgery, Leiden University, Leiden, The Netherlands; d Research Department, Kliniek ViaSana, Mill, The Netherlands; e Department of Health Sciences, Faculty of Science, Vrije Universiteit Amsterdam, Amsterdam, The Netherlands; f Department of Epidemiology and Data Science, Amsterdam UMC Location Vrije Universiteit, Amsterdam, The Netherlands; g Department of Methodology, Amsterdam Public Health Research Institute, Amsterdam, The Netherlands

**Keywords:** PROMs, PROMIS, Psychometric properties, Total knee arthroplasty

## Abstract

**Background:**

Traditionally used PROMs evaluating total knee arthroplasty (TKA) have large measurement error and limited measurement range. Patient-Reported Outcomes Measurement Information System (PROMIS) has theoretical possibilities to overcome these limitations. This study evaluates the psychometric properties of PROMIS vs legacy PROMs in TKA patients.

**Methods:**

210 patients were included from 3 orthopaedic departments. Patients completed a questionnaire twice in a 2-week interval, including 2 PROMIS Computer Adaptive Tests (CATs), assessing Physical Function (PF) and Pain Interference (PI), 5 PROMIS short forms (SF) (PF-SF8b, PF-SF10a, PF-SF-20a, PI-SF8a, PI-1a) and 6 legacy PROMs (KOOS, KOOS-PS, WOMAC, OKS, 2 numeric rating scales). Reliability, measurement precision, smallest detectable change (SDC), construct validity, burden and extreme scores were investigated.

**Results:**

All PROMIS CATs, SFs, and legacy PROMs showed adequate test–retest reliability. PROMIS CATs and SFs showed sufficient construct validity. Regarding PF, the SDC varied between 2.8-6.1 for PROMIS and 7.3-19.9 for legacy PROMs. PROMIS CAT and SFs showed no extreme scores using 5-20 items, legacy PROMs showed 1.2%-3% extreme scores using 7-17 items. Concerning pain, the SDC varied between 2.8-6.1 for PROMIS and 22.5-35.4 for legacy PROMs. PROMIS showed 0%-9.5% extreme scores using 5-8 items and legacy PROMs 8.7%-19.1% using 1-9 items.

**Conclusions:**

PROMIS CAT and SFs seem more efficient assessing PF in TKA compared to legacy PROMs by offering reduced burden and measurement error, and minimizing extreme scores. PROMIS PI-8a seems most suitable in measuring pain without extreme scores. This study supports a shift from legacy PROMs toward PROMIS in TKA patients.

## Introduction

Patients' perspectives on total knee arthroplasty (TKA) effectiveness are evaluated using patient-reported outcome measures (PROMs). The traditionally used (‘legacy’) PROMs evaluating TKA have several disadvantages regarding measurement properties. The measurement error is often too large for reliable use in individual patients [1,2]. Other challenges include limited reliability and responsiveness [2]. Therefore, these PROMS are not optimal for use in the consultation room with the individual patient. More reliable measurements can ensure better patient monitoring and improve quality of care [3].

An innovation in PROMs is the possibility of using Computer Adaptive Tests (CATs). CAT selects items from item banks, which are collections of questions calibrated on an underlying measurement scale, using Item Response Theory (IRT) modeling. The Patient-Reported Outcomes Measurement Information System (PROMIS) is the largest and most extensively validated system of CATs for measuring self-reported health [4]. PROMIS CAT is a dynamic form of testing that adjusts the items questioned to a patient based on their previous responses. This makes the process more responsive to the patient’s specific health condition and minimizes the response burden [5,6]. Patients need to complete, on average, only 4-7 items to get a reliable score [7]. Furthermore, the item banks cover the entire construct width and therefore, floor and ceiling effects likely do not arise.

In addition to CAT, all PROMIS measures are also available in static short forms (SFs), containing a fixed set of items from the item bank. SFs may be used when CAT is not yet feasible. SF and CAT scores are directly comparable since the scores are expressed on the same metric (scale). These SFs outperformed legacy PROMs in patients with osteoarthritis regarding reliability and measurement range. However, PROMIS CAT performed superiorly over SFs for most measurement properties and burden [7,8].

PROMIS is extensively used in the orthopaedic field. Most studies showed a sufficient correlation between PROMIS and legacy PROMs [9]. However, little is published on the comparative psychometric properties between PROMIS and legacy PROMs, with only a few studies in TKA patients [[9], [10], [11]].

This study aimed to compare the performance of PROMIS CATs and PROMIS SFs to legacy PROMs, focusing on function and pain in TKA patients. The reliability, measurement precision, smallest detectable change (SDC), construct validity, extreme scores and burden of legacy PROMs will be compared head-to-head to PROMIS CATs and PROMIS SFs in TKA patients.

## Material and methods

### Design

This is a multicenter test–retest study at 3 high-volume TKA orthopaedic departments in the Netherlands (St. Antonius Hospital in Utrecht, Kliniek ViaSana in Mill, OLVG in Amsterdam). Institutional review board approval was obtained for each participating center.

### Study participants

Adult patients were eligible for study inclusion either preoperatively, while on the waiting list for a primary TKA, or postoperatively. This ensures variability in scores. From the study start date, consecutive patients awaiting surgery attending their 6- or 12- month postoperative follow-up were included in the study. Therefore, each patient completed a single test–retest assessment. Excluded were patients unable to independently fill out questionnaires or without internet facilities. This study was nested in the regular PROM administration. Eligible patients were approached during routine PROM administration in each hospital and asked to digitally sign informed consent. A sample size of 100 patients was considered very good for assessing measurement properties [12]. Each hospital included a minimum of 50 patients distributed over the measurement points.

### Procedure

Patients were asked to complete an online questionnaire twice at a 2-week interval. This design ensures no (large) changes in pain and function, and enables the assessment of reliability, including the SDC. Questionnaires were emailed through a web-based platform (OnlinePROMS, Interactive Studios‘s-Hertogenbosch, the Netherlands). This certified (ISO27001; NEN7510) OnlinePROMs platform is linked to the Dutch-Flemish Assessment Center CAT software. Missing data were not allowed, as the system is designed to prevent incomplete submissions. A maximum of 2 automatic reminders were sent every 2 days after the first invitation when the patient did not respond.

### Measurement instruments

Patients’ physical function (PF), pain intensity, and pain interference (PI) were measured using 2 Dutch-Flemish PROMIS CATs, 5 Dutch-Flemish PROMIS short forms, and 6 legacy PROMs (Table 1). The retest questionnaire included the same PROMIS CATs, PROMIS SFs, and legacy PROMs.

**Table 1:** Characteristics of included measurement instruments.

Measures	Construct / definition	Items	Response options	Score	Recall
PROMIS measures					
PROMIS CAT PF (PROMIS PF, v1.2; [12,13])	Functioning of one’s upper extremities (dexterity), lower extremities (walking or mobility), and central regions (neck, back), as well as instrumental activities of daily living	Min 3 Max 12	5-point Likert	T-score^a^, higher T-score indicating better PF	-
PROMIS PF SF20a (v1.2; [14,15])	Functioning of one’s upper extremities (dexterity), lower extremities (walking or mobility), and central regions (neck, back), as well as instrumental activities of daily living	20	5-point Likert	T-score^a^, higher T-score indicating better PF	-
PROMIS PF SF10a (v1.2; [14,15])	Functioning of one’s upper extremities (dexterity), lower extremities (walking or mobility), and central regions (neck, back), as well as instrumental activities of daily living	10	5-point Likert	T-score^a^, higher T-score indicating better PF	-
PROMIS PF SF8b (v1.2; [14,15])	Functioning of one’s upper extremities (dexterity), lower extremities (walking or mobility), and central regions (neck, back), as well as instrumental activities of daily living	8	5-point Likert	T-score^a^, higher T-score indicating better PF	-
PROMIS CAT Pain Interference (PROMIS-PI, v1.1; [16])	Consequences of pain on relevant aspects of one’s life	Min 3 Max 12	5-point Likert	T-score^a^, higher T-score indicating more pain	Last 7 d
PROMIS PI SF8a (v1.1; [17,18])	Consequences of pain on relevant aspects of one’s life	8	5-point Likert	T-score^a^, higher T-score indicating more pain	Last 7 d
PROMIS Pain Intensity 1a (v1.0; [19,20])	How much a person hurts	1	11-option numeric rating scale	0 (no pain) - 10 (worst thinkable pain)	Last 7 d
Legacy PROMs					
KOOS; [21]	5 subscales - Pain - Symptoms - Function in daily living (ADL) - Sport and recreation Function (Sport/Rec) Quality of Life (QOL)	42 - 9 - 7 - 17 - 5 - 4	5-point Likert	0 (indicating extreme symptoms) - 100 (indicating no symptoms)	Last week
KOOS-PS; [22]	PF	7	5-point Likert	Raw scores were converted (0-100, 0 indicating extreme knee symptoms) (31)	Last week
WOMAC; [23]	3 subscales: - Pain - Stiffness Function	24 - 5 - 2 - 17	5-point Likert	Raw scores were converted (0-100, 0 indicating extreme knee symptoms) (31)	Last 48 h
Oxford Knee Score [24]	Function and pain	12	5-point Likert	0-48 (0 indicating the worst, 48 the best outcome)	Past 4 wk
NRS Pain activity (No reference available)	Pain during activity	1	11-option numeric rating scale	0-100 (0 indicating the worst, 100 the best outcome)	Last wk
NRS Pain rest (No reference available)	Pain at rest	1	11-option numeric rating scale	0-100 (0 indicating the worst, 100 the best outcome)	Last wk

NRS, numeric rating scale; ADL, activities of daily living; WOMAC, Western Ontario and McMaster Universities Osteoarthritis Index.

a T-score 50 represents the average score of the general population, SD of 10.

The 2 PROMIS CAT measures included were the PROMIS v1.2 CAT Physical Function [13,17] and PROMIS v1.1 CAT PI [14]. PROMIS CAT identifies the most relevant questions from its item banks based on responses to both initial and follow-up questions. This process continues until a specified level of reliability is reached. The CATs automatically stopped when a standard error (SE) of 2.2 was achieved, corresponding to 95% reliability, or when a maximum of 12 items were answered. The CAT software used in this study utilized maximum likelihood estimation, which means that determining the T-score and SE requires variation in item responses. When patient responses were uniform on the CAT (all positive or all negative responses), the SE could not be estimated, leading to imputed T-scores (these scores were set to 0 or 100).

Furthermore, 3 PROMIS SFs measuring PF were included (PROMIS Physical Function SF8b, SF10a, and SF20a [15,18]. In addition, 1 PROMIS SF measuring PI (SF8a [14,19]) and the single-item PROMIS Pain Intensity 1a [20,21] were included.

PROMIS uses a T-score metric with an average score of 50 and a standard deviation (SD) of 10. A score of 50 reflects the average performance of the general population. A higher PROMIS T-score indicates a greater degree of the measured concept (such as better function or more pain).

The questionnaire included the following legacy PROMs for PF and pain: the Knee injury and Osteoarthritis Outcome Score (KOOS [22]), KOOS-Physical function Shortform (KOOS-PS [23]), Western Ontario and McMaster Universities Osteoarthritis Index [24], Oxford Knee Score [25] and 2 numeric rating scales measuring pain during activity and pain in rest (Table 1). The legacy PROMs were developed under a Classical Test Theory (CTT) model. Characteristics of these legacy PROMs can be found in Table 1.

Finally, patient characteristics (sex, age, and date of surgery) were collected.

### Data analysis

Reliability, measurement precision (SE of measurement (SEM), SDC, construct validity, burden and extreme scores were compared between PROMIS CATs, PROMIS SFs, and legacy PROMS.

#### Test–retest reliability

The intraclass correlation coefficient (ICC) was calculated to assess test–retest reliability for each of the PROMIS CATs, PROMIS SFs and legacy PROMs with total scales or subscales. The ICC was calculated using a 2-way random-effects model for absolute agreement, following the formula: $agreement=\frac{\sigma_{p}^{2}}{\sigma_{p}^{2}+\sigma_{m}^{2}+\sigma_{e}^{2}}$. In this formula $\sigma_{p}^{2}$ is the variation between patients, $\sigma_{m}^{2}$ is the variation between measurements and $\sigma_{e}^{2}$ is the random error variance. An ICC value of 0.70 or higher was considered indicative of 'sufficient' test–retest reliability [26].

#### Measurement precision

Measurement precision was assessed by calculating the SEM. Since the possible range of the score differs per outcome measure and is important for interpreting the SEM, both the SEM and the range of the score are presented. The PROMIS CATs and SFs were created using an IRT model. In this framework, each T-score is associated with a SEM = SE(T-score). Measurement error is not constant across the scale, implying that each score (and each consequently each patient) has a unique SEM value. The PROMIS CAT software automatically computes the SEM for each patient's score. The legacy PROMs are developed under a CTT model, assuming all scores have the same SEM. The SEM for the legacy PROMs was calculated using the formula: $SEMagreement=\sqrt{\sigma_{m}^{2}\;{+\sigma }_{e}^{2}}$.

#### Smallest detectable change

Minor fluctuations in scores may reflect true changes in the patient’s condition, or may result from measurement errors. The SDC was calculated, defined as the minimum change in score above which there is at least a 95% chance that a real change has occurred [26]. The SDC was calculated using the formula: SDC=1.96∗√2∗SEM. For PROMIS CATs and SFs (based on IRT) the individual SEM for both the test T-score and the retest T-score was used in the calculation: $(SDC=1.96*\sqrt{SE_{1}^{2}+SE_{2}^{2}}$). They yield a distinct SDC value for each individual patient. Consequently, the mean and range of T-scores are presented for each PROMIS CAT or SF.

Given the variations in measurement scales, there is no widely accepted approach for comparing SEM or SDC of measurement instruments based on differing underlying theories (IRT vs CTT). We described these difficulties in a previous study by our research group (Braaksma et al., 2024). Because of these difficulties, this study reports the number of points of the SEM and SDC within the instrument’s specific scale. The reported SEM and SDC cannot be directly compared between measurement instruments but are considered useful for interpreting scores of a given measurement instrument.

#### Construct validity

Construct validity is defined as the degree to which a measure’s scores are consistent with hypotheses based on the assumption that the intended construct is validly measured by the PROM [27]. To examine construct validity of PROMIS CAT and PROMIS SFs, hypotheses were formulated a priori, based on expected correlations between the PROMIS CAT and PROMIS SFs, and legacy PROMs for each construct (PF and pain). (1). A high correlation was expected between instruments measuring the same construct (eg, PROMIS PF and KOOS-PS). (2). Moreover, a high correlation was expected between PROMIS CATs and SFs in the construct pain with legacy PROMs evaluating physical functioning. And vice versa; a high correlation was expected between PROMIS CATs and SFs in the construct PF with legacy PROMs evaluating pain. It has been shown that as pain levels increase, an individual’s physical functioning typically declines. This reinforces the anticipated strong correlation [16,17,28,29]. Additionally, it was expected that correlations among measurement instruments evaluating the same construct (eg, PROMIS PF and KOOS-PS) would exceed those among instruments assessing different but related constructs (eg, PROMIS PF and legacy PROMs measuring pain, stiffness or quality of life).

To evaluate construct validity, Pearson's correlations were calculated between the PROMIS CATs and SFs, as well as legacy PROMs, resulting in a total of 91 unique predetermined hypotheses. Construct validity was considered adequate if at least 75% of the findings confirmed the proposed hypotheses [12]. If a correlation was greater than 0.7, the hypotheses was accepted.

#### Feasibility

##### Burden

Burden was defined as the number of items per instrument to asses physical functioning and pain.

#### Interpretability

##### Range of scores

Extreme scores were defined as minimum or maximum possible scores. The percentage of patients with extreme scores was reported for each measurement instrument.

## Results

**Figure 1:**
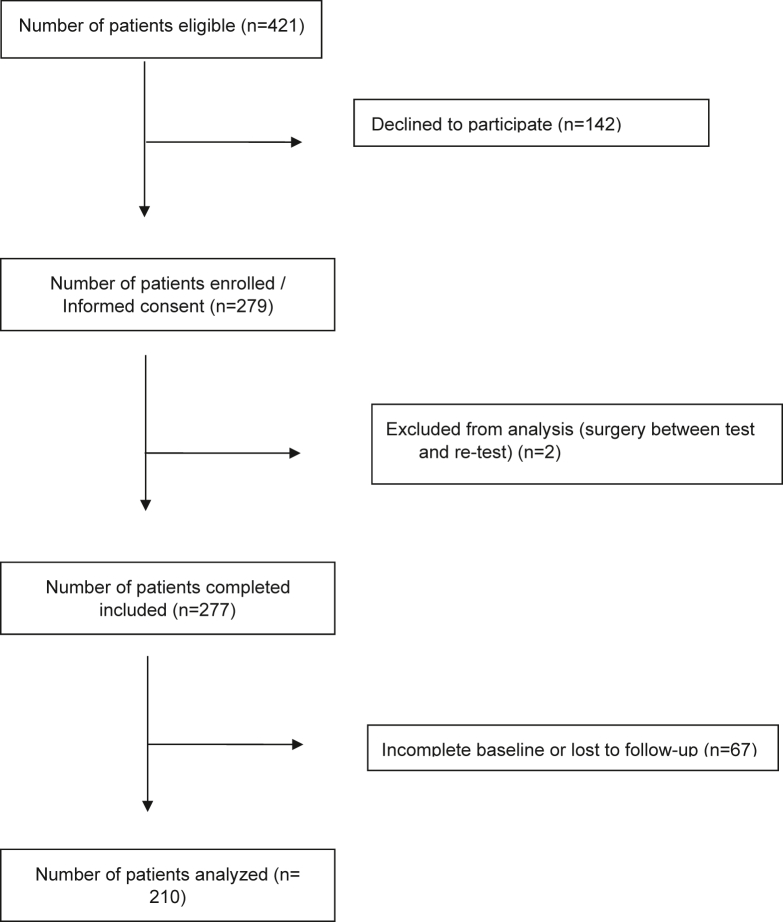
Flowchart of inclusion.

A total of 210 participants were included. Figure 1 presents the flowchart of inclusion. The mean age was 68.3 years (SD 7.7), ranging from 45 to 85 years, with 51.9% being men (n = 109). The mean time interval between test and retest was 8 days (SD 2). Table 2 provides details on the mean (SD, range) scores of all PROMIS CATs, PROMIS SFs, and legacy PROMs.

**Table 2:** Average scores and range per measurement point.

Measures	Mean (SD; range)	Retest	6 mo (n = 67)	Retest	12 mo (n = 28)	Retest
	Preoperative (n = 115)					
	Baseline		Baseline		Baseline	
PROMIS CAT PF	37.4 (5.9; 18.7-53.1)	37.4 (6.4; 12.6-52.5)	45 (6.3; 23.8-54.9)	45.6 (6.3; 28.2-54.8)	45.1 (5.6; 34.4-53.6)	44.5 (5.9; 30.2-55)
PROMIS SF PF 20a	36.3 (5.5; 20.6-49.2)	36.2 (5.8;15.3-47.5)	43.1 (6.5;18.3-62.7)	42.9 (6.1;20.1-54.9)	43.3 (6.8;32.7-62.7)	43.9 (7.24;30.7-57)
PROMIS SF PF 10a	35.6 (5.3; 22-49.4)	35.7 (5.7;13.5 -47.9)	43.3 (6.7;20.9-61.9)	43 (6.2; 23.1-55.8)	43.1 (7.05;29.4-61.9)	43.9 (7.4;31-61.9)
PROMIS SF PF 8b	36.1 (5.6; 20.9-59.7)	36.1 (5.7;20.9-52.5)	43.8 (7.3; 20.9-59.7)	44.1 (7.4;24.4-59.7)	45.8 (8.4;31.1-59.7)	45.1 (8.1;27.9-59.7)
PROMIS CAT Pain interference	63.6 (5.5; 50.4-81)	63.6 (5.4; 52-76.6)	55.4 (6.9;44.6-75)	54.7 (6;44.6-69.4)	55.7 (8.2;44.6-67.6)	55.9 (7;44.7-67.6)
PROMIS SF Pain interference 8a	62.1 (5.9; 40.7-77)	61.8 (5.7;48-77)	51.5 (8.1; 40.7-77)	50.2 (8.8; 40.7-68.4)	51.6 (8.9; 40.7-66.9)	49.9 (8.7; 40.7-64.1)
PROMIS SF Pain intensity 1a	6 (2.1;1-10)	6.2 (2.1; 1-10)	2.7 (2.3;0-9)	2.5 (2.3;0-8)	2.3 (2.3; 0-7)	2.4 (2.2; 0-7)
KOOS	45.7 (17.3;4.2-82.1)	44.3 (16.9 (2.4-82.7)	72.3 (16.5;5.4-100)	71.8 (15.7;31-100)	74.5 (16.2;36.9-94.6)	74.4 (16.6;44.6-100)
KOOS-PS	48.1 (16.1;14.8-100)	50.6 (16.7; 18.6-100)	30.1 (12.4; 0-62)	31.3 (12.1; 0-57.9)	29.5 (11.9; 10.5-51.2)	30.7 (14.2; 0-62)
KOOS-Symptoms	54.2 (21.7; 0-89)	52.8 (19.3; 0-96.4)	70.3 (17.1; 10.7-100)	71.1 (15.1; 39.3-100)	74.7 (17.2; 39.3-100)	76.4 (15.2; 46.4-100)
KOOS-QOL	29.6 (16.2; 0-81.3)	30 (15.9; 0-81.3)	61 (19.9; 0-100)	60.7 (19.3; 6.3-100)	64.3 (22.8; 6.3-100)	63.8 (22.6; 12.5-100)
KOOS-Sport/Recr	19.5 (17.1;0-65)	20.2 (24.1; 0-100)	44.9 (24.3; 0-100)	42.5 (27.4; 0-100)	45.5 (22.1; 5-85)	43.9 (27.9;0-100)
KOOS-ADL	53.2 (20.5; 5.9-91.1)	49.9 (19.9; 0-88.2)	80.6 (16.4; 7.4-100)	79.9 (15; 32.3-100)	82.2 (16.5;38.2-100)	81.3 (16.4; 48.5-100)
KOOS-Pain	46.9 (19.7;0-83.3)	46.6 (18.8; 0-86.1)	78.6 (19.8; 0-100)	78.3 (17.8;19.4-100)	80.5 (18.3; 38.9-100)	81.7 (16.9; 47.2-100)
OKS	24.9 (8.1; 5-43)	24.3 (8.6; 1-42)	36.4 (8.3; 10-48)	36.6 (8.1; 12-47)	38.6 (7.9; 20-48)	38.4 (7.5; 24-48)
WOMAC	52.3 (19.9; 7.3-88.5)	49.4 (19.1;0-87.5)	79.7 (16.5; 5.2-100)	79 (15.3; 32.3-100)	81.7 (16.5; 39.6-100)	81.2 (15.8; 49-100)
WOMAC-Pain	52.9 (21.2; 0-95)	50.9 (19.2; 0-90)	82.7 (18.5; 0-100)	81.6 (17.2; 30-100)	84.3 (18; 35-100)	85 (15.6;50-100)
WOMAC-Stiffness	43.7 (25.3; 0-100)	41.7 (22.5; 0-100)	64.4 (22.3;0-100)	61.8 (22.6; 12.5-100)	70.5 (22.4; 12.5-100)	71 (19.6; 25-100)
WOMAC-Function	53.2 (20.5; 5.9-91.2)	49.9 (19.9; 0-88)	80.6 (16.4;7.4-100)	79.9 (15; 32.4-100)	82.2 (16.5; 38.2-100)	81.3 (16.4; 48.5-100)
NRS Pain Activity	67.3 (22.4; 0-100)	65.9 (22.6; 0-100)	31.9 (24.8; 0-80)	31 (26; 0-90)	29.3 (27.9;0-80)	26.1 (25.3;0-80)
NRS Pain Rest	45.1 (25.2; 0-100)	45.4 (25.5;0-100)	20.5 (22; 0-70)	21.8 (24.4;0-90)	19.2 (24.2;0-80)	20 (21.6;0-70)

n, number of patients; MIC, minimally important change; QOL, quality of life; Sport/Recr, sports/recreation; ADL, activities of daily living; OKS, Oxford Knee Score; WOMAC, Western Ontario and McMaster Universities Osteoarthritis Index.

### Test–retest reliability

Results showed evidence for sufficient test–retest reliability for all PROMIS CATs, PROMIS SFs, and legacy PROMs (ICCs between 0.74 and 0.94, Table 3).

**Table 3:** The mean SEM, SDC, burden, ICC, and the percentage of patients with minimum and maximum scores of PROMIS CAT, PROMIS SFs, and legacy PROMs.

Measures	SEM mean (range)	SDC mean (range)	ICC agreement (95% CI)	Burden (mean) number of items	Minimum score (%)	Maximum score (%)	Score range
PROMIS instruments							
PROMIS CAT PF	2.1 (1.9-2.2)	5.7 (5.3-6.1)	0.90 (0.87-0.92)	5.2	0	0	12.6-55
PROMIS PF SF20a	1.6 (1.3-5.7)	4.4 (3.6-13.2)	0.91 (0.88-0.93)	20	0	0	15.3-62.7
PROMIS PF SF10a	2 (1.7-5.9)	5.6 (4.7-13.9)	0.89 (0.86-0.92)	10	0	0	13.5-61.9
PROMIS PF SF8b	1.9 (1.5-5.9)	5.4 (4.2-16.4)	0.90 (0.87-0.92)	8	0	0	20.9-59.7
PROMIS CAT PI	2.1 (1.7-3.6)	6.1 (3.6-16.4)	0.78 (0.72-0.83)	4.8	7.7	0	44.6-81
PROMIS PI SF8a	2.1 (1.3-5.9)	6.1 (3.6-16.4)	0.84 (0.80-0.88)	8	0	0	40.7-77
PROMIS Pain Intensity 1a	1	2.8	0.87 (0.84-0.90)	1	9.3	0.2	0-10
Legacy PROMs							
KOOS	5.4	15.1	0.94 (0.92-0.95)	42	0.3	0	2.4-100
KOOS-PS	6.9	19	0.85 (0.80-0.88)	7	0.9	0.9	0-100
KOOS-Symptoms	7.2	19.9	0.88 (0.85-0.91)	7	0.3	2.8	0-100
KOOS-QOL	7.1	19.7	0.91 (0.89-0.93)	4	0.3	0	0-100
KOOS-Sport/Recr	13.3	37	0.74 (0.67-0.80)	5	38.4	4.3	0-100
KOOS-ADL	7.2	19.9	0.91 (0.88-0.93)	17	0.5	3.8	0-100
KOOS-Pain	8.1	22.5	0.89 (0.86-0.92)	9	0.3	8.4	0-100
OKS	2.6	7.3	0.94 (0.92-0.95)	12	0	1.2	7.9-48
WOMAC	6.7	18.5	0.92 (0.89-0.94)	24	0.2	2.1	0-100
WOMAC - Pain	8.4	23.4	0.88 (0.85-0.91)	5	0.9	10.2	0-100
WOMAC - Stiffness	11.9	32.9	0.79 (0.73-0.84)	2	2.8	6.4	0-100
WOMAC - Function	7.2	19.9	0.91 (0.88-0.93)	17	0.2	2.8	0-100
NRS pain – Activity	12.2	33.9	0.83 (0.79-0.87)	1	9.4	2.1	0-100
NRS pain - Rest	12.8	35.4	0.78 (0.72-0.83)	1	18.4	.7	0-100

CI, confidence interval; QOL, quality of life; Sport/Recr, sports/recreation; ADL, activities of daily living; NRS, numeric rating scale; OKS, Oxford Knee Score; WOMAC, Western Ontario and McMaster Universities Osteoarthritis Index.

### Measurement precision

The SEM of PROMIS CATs and SFs ranged between 1 and 2.1. The SEM of the legacy PROMs varied between 2.6 and 13.3. For legacy PROMs which are scaled from 0 to 100, the lowest SEM for pain and PF was observed for respectively the KOOS pain (SEM 8.1) and the KOOS-PS (SEM 6.9) (Table 3).

### Smallest detectable change

The SDC for PROMIS CATs and SFs varied between 2.8 and 6.1 and for legacy PROMs between 7.3 and 37. Regarding PF, the lowest SDC for PROMIS CAT and SF was found using the PROMIS PF SF20a (SDC 4.4). The lowest SDC among the legacy PROMs with a 0-100 scale was the KOOS-PS (SDC 19). Regarding the construct pain, the SDC of PROMIS CAT and SF8a was equal (SDC 6.1). The lowest SDC among the legacy PROMs with a 0-100 scale was the KOOS-pain (SDC 22.5). The SDC of the 3 numeric rating scales measuring pain (PROMIS Pain Intensity 1a en 2 legacy PROMs) was respectively 2.8 (scale 0-10), 33.9 and 35.4 (scale 0-100).

### Construct validity

The results indicated sufficient construct validity for all PROMIS CATs and PROMIS SFs (Table 4). 77% to 100% of the results were in accordance with the predefined hypotheses. All measurement instruments measuring PF (PROMIS CAT-PF, PROMIS SF PF 20a, 10a, and 8b) correlated highly (mean Pearson’s r 0.81-0.83) with legacy instruments measuring PF and legacy instruments measuring pain (mean Pearson’s r 0.71-0.73), as hypothesized. All instruments evaluating pain (PROMIS CAT-PI, PROMIS SF PI8a, PROMIS Pain intensity 1a) correlated highly with legacy instruments measuring pain (mean Pearson’s r 0.72-0.84) and PF (mean Pearson’s r 0.83-0.84). The correlations among the measurement instruments evaluating the same constructs exceeded those among instruments assessing different but related constructs.

**Table 4:** Pearson’s r for correlations between construct of PROMIS CATs, PROMIS SFs, and legacy PROMs (n = 210).

Domain	PF						Pain					Other				Total
Measurement instrument	OKS	KOOS-PS	KOOS ADL	WOMAC function	WOMAC total	MEAN^a^	NRS pain activity	NRS pain rest	WOMAC pain	KOOS pain	MEAN^a^	KOOS sport/rec	KOOS symptoms	WOMAC stiffness	KOOS QOL	% of findings aligned with hypotheses proposed hypotheses
PROMIS CAT Physical Functioning	**0.86**	**−0.74**	**0.82**	**−0.82**	**0.82**	0.81	**−0.72**	−0.59	**0.77**	**0.77**	0.71	**0.60**	**0.62**	**0.61**	0.76	**85**
PROMIS SF Physical Functioning 20a	**0.86**	**−0.77**	**0.84**	**0.84**	**0.83**	0.83	**−0.70**	−0.59	**0.76**	**0.77**	0.71	**0.62**	**0.66**	**0.62**	0.74	**85**
PROMIS SF Physical Functioning 10a	**−0.86**	**0.75**	**−0.84**	**−0.84**	**−0.85**	0.83	−0.61	0.65	**−0.81**	**−0.84**	0.73	**−0.64**	**−0.67**	**−0.65**	−0.83	**77**
PROMIS SF Physical Functioning 8b	**0.86**	**−0.74**	**0.83**	**0.83**	**0.83**	0.82	**0.73**	−0.59	**0.78**	**0.78**	0.72	**0.61**	**0.60**	**0.62**	0.78	**85**
PROMIS CAT Pain Interference	**−0.86**	**0.72**	**−0.80**	**−0.80**	**−0.81**	0.84	**−0.71**	−0.62	**−0.76**	**−0.78**	0.72	**−0.57**	**−0.63**	**−0.60**	−0.82	**85**
PROMIS SF Pain interference 8a	**−0.86**	**0.75**	**−0.84**	**−0.84**	**−0.85**	0.83	**0.77**	0.65	**−0.81**	**−0.84**	0.77	**−0.64**	**−0.67**	**−0.65**	−0.83	**85**
PROMIS SF Pain intensity 1a	**−0.84**	**0.75**	**−0.84**	**−0.84**	**−0.85**	0.84	**0.87**	**0.79**	**−0.84**	**−0.85**	0.84	**−0.61**	**−0.64**	**−0.65**	**0.80**	**100**

Bold values represent hypotheses in line with expectation.

QOL, quality of life; Sport/Recr, sports/recreation; ADL, activities of daily living; OKS, Oxford Knee Score; WOMAC, Western Ontario and McMaster Universities Osteoarthritis Index.

a The mean correlation was calculated per construct (physical function and pain) per measurement instrument. It was expected that the correlation per PROMIS instrument with the mean correlation of legacy PROMs measuring the same construct (such as PROMIS PF compared to the mean correlation of the legacy PROMs measuring physical function) would exceed those among instruments assessing different but related constructs (such as PROMIS PF compared to the mean correlation of legacy PROMs measuring pain, stiffness or quality of life).

### Feasibility

For the PROMIS CAT PF and PI, approximately 5 items were necessary to achieve the predefined reliability threshold (Table 3). Based on previous research, the average completion rate for PROMIS CAT instruments is approximately 5 items per minute(Cella et al., 2010). The 3 PROMIS SFs for assessing PF consisted of 8, 10, and 20 items respectively. The number of items of the legacy PROMs measuring PF varied from 1 to 42. Regarding the construct pain, the included PROMIS SF measuring PI contained 8 items. PROMIS Pain Intensity contained a single item. There were 2 single-item pain legacy PROMs included and 2 subscales of legacy PROMs measuring pain consisting of 5 and 9 items respectively.

### Interpretability

PROMIS Physical Function CAT and SFs did not present any minimum or maximum scores. Legacy PROMs measuring PF had 1.2%-3.2% extreme scores. Regarding pain, 8.2% of the patients scored at the best end of the scale for the PROMIS PI CAT, 0% for the PROMIS PI SF8a and 9.5% for the PROMIS Pain Intensity. For legacy PROMs measuring pain, extreme scores were observed in 8.7% to 42.7% of patients (Table 2).

## Discussion

This study showed that PROMIS CAT PF offers a more efficient alternative to legacy PROMs in assessing PF in TKA patients, without compromising measurement quality. Both PROMIS CAT and SF measuring PF avoid extreme score distributions. Regarding the construct pain, the PROMIS PI 8a was the only measurement instrument without extreme scores with an average burden of 8 items and sufficient reliability and construct validity.

PROMIS CATs en SFs, particularly CAT PF, may be better suited for clinical monitoring and decision-making in TKA patients due to their reduced burden lack of extreme scores, compared to legacy PROMs. The PROMIS PF CAT and SFs showed no extreme scores. Especially when there is a need to measure patients with extremely poor function or almost no symptoms, it is important that the instruments can measure at the extremes of the scale. These findings are consistent with those of Dhollander et al. (2025), confirming that PROMIS PF demonstrates excellent precision, responsiveness, and feasibility in TKA patients [11]. Our multicenter design, test–retest reliability and inclusion of PROMIS Short Forms and the Oxford Knee Score provide additional evidence on the broader applicability and comparative performance of PROMIS instruments. This study showed the superior performance of PROMIS CAT PF relative to PROMIS SF PF, previously described by Fries et al. [8]. However, when the implementation of CAT remains impractical the PROMIS PF SF8b is the best alternative. Although it consists of an average of 3 more items than the CAT, the SEM and SDC are comparable. Interestingly, the PROMIS SF20a has a slightly lower SDC and SEM. This is probably attributed to the higher number of items questioned, which is also the disadvantage of this SF.

As for the construct pain, only the PROMIS PI 8a could measure without extreme scores. All single item PROMs measuring pain (PROMIS Pain intensity 1a and both legacy PROMs) and the legacy PROMs were not good at measuring at the end of the scale (>8% extreme scores). Regarding the construct pain, PROMIS CAT and SF had a comparable SDC and SEM.

This study confirmed previously reported strong correlations between PROMs assessing pain and PF [16,17,28,29]: higher pain levels correlate with lower levels of PF. It could be hypothesized that measuring both PI and PF might not be necessary in patients who experience pain.

The strength of this study was the thorough designed prospective multicenter study across the country, which may lead to generalizability of the results. A methodological limitation of our study is the use of the outdated maximum likelihood estimation, which may have contributed to a higher percentage of extreme scores in the PROMIS PI CAT. It is expected that when the standard expected a priori method will be used, there will be less extreme scores. Another methodological challenge was the interpretation of the SDC and SEM. This remains complex, as comparisons across different scales of CTT and IRT-based measurement instruments cannot be made adequately. We present the SDC and SEM values per measurement instrument, accompanied by the range of the scales for interpretation purposes. Other methods for bypassing this problem were previously described, but no consensus exist regarding the best approach [28].

To facilitate the selection of the most appropriate measurement instruments for clinical practice, future research should focus on comparing the minimal important change and responsiveness between PROMIS instruments and legacy PROMs in TKA patients. In addition to pain and PF, other domains as mental health could be assessed.

## Conclusions

Both PROMIS CAT and SFs seem most efficient for assessing patient-reported PF in TKA, compared to legacy PROMs. This by offering reduced burden and measurement error, and minimizing the occurrence of extreme scores. The PROMIS PI 8a seems most efficient for assessing pain in TKA patients, minimizing extreme scores. The results of this study can facilitate better patient monitoring and decision-making in TKA patients. These findings may suggest a need for a reevaluation of routine outcome measurement using legacy PROMs.

## Conflicts of interest

C.B. Terwee is past president of the PROMIS Health Organization and head of the Dutch-Flemish PROMIS National Center and receives royalties from the textbook Measurement in Medicine

R.W. Poolman receives research support from Link as a principal investigator, is a member of the editorial board of OrthoEvidence, and is a board member of the Dutch Orthopaedic Society.

The other authors declare no potential conflicts of interest.

For full disclosure statements refer to https://doi.org/10.1016/j.artd.2026.101964.

## CRediT authorship contribution statement

**Christel Braaksma** Writing – review & editing, Writing – original draft, Visualization, Project administration, Methodology, Investigation, Funding acquisition, Formal analysis, Data curation, Conceptualization. **Nienke Wolterbeek:** Project administration, Methodology, Investigation, Funding acquisition, Data curation, Conceptualization. **Maurits Remmelt Veen:** Writing – review & editing, Methodology, Funding acquisition, Conceptualization. **Rudolf Wilhelm Poolman:** Writing – review & editing, Validation, Supervision, Methodology, Funding acquisition, Conceptualization. **Yvette Pronk:** Writing – review & editing, Supervision, Project administration, Methodology, Investigation, Funding acquisition, Data curation, Conceptualization. **Ariena Jorien Rasker:** Writing – review & editing, Validation, Project administration, Methodology, Investigation, Formal analysis, Data curation, Conceptualization. **Raymond Willem Jozef Gerardus Ostelo:** Writing – review & editing, Validation, Supervision, Methodology, Investigation. **Caroline Barbara Terwee:** Writing – review & editing, Validation, Supervision, Software, Resources, Methodology, Investigation, Funding acquisition, Formal analysis, Data curation, Conceptualization.

## Appendix A Supplementary data

Conflict of Interest Statement for Braaksma

Conflict of Interest Statement for Terwee

Conflict of Interest Statement for Veen

Conflict of Interest Statement for Wolterbeek

Conflict of Interest Statement for Poolman

Conflict of Interest Statement for Ostelo

Conflict of Interest Statement for Rasker

Conflict of Interest Statement for Pronk
