# Supplementary material for: A Comparison of the Psychometric Properties of PROMIS Computer Adaptive Tests and Short Forms Vs Legacy Patient-Reported Outcome Measures in Total Knee Arthroplasty Patients
Source: Arthroplast Today. 2026 Mar 6;38:101964. doi: 10.1016/j.artd.2026.101964 (PMC12993152; doi:10.1016/j.artd.2026.101964)
Supplement: Conflict of Interest Statement for Braaksma [file mmc1.docx]

# INDIVIDUAL CONFLICT OF INTEREST STATEMENT

***American Association of Hip and Knee Surgeons***

(Adopted from the American Academy of Orthopaedic Surgeons disclosure statement)

The following form **must be filled out completely and submitted by each author (example, 6 authors, 6 forms).**

**All items require a response. If there is no relevant disclosure for a given item, enter "*None*.”**

**Manuscript Title**

**A comparison of the psychometric properties of PROMIS computer adaptive tests and short forms versus legacy patient-reported outcome measures in total knee arthroplasty patients**

1. Royalties from a company or supplier (The following conflicts were disclosed)

none

2. Speakers bureau/paid presentations for a company or supplier (The following conflicts were disclosed)

none

3A. Paid employee for a company or supplier (The following conflicts were disclosed)

none

3B. Paid consultant for a company or supplier (The following conflicts were disclosed)

none

3C. Unpaid consultants for a company or supplier (The following conflicts were disclosed)

None

4. Stock or stock options in a company or supplier (The following conflicts were disclosed)

None

5. Research support from a company or supplier as a Principal Investigator (The following conflicts were disclosed)

None

6. Other financial or material support from a company or supplier (The following conflicts were disclosed)

None

7. Royalties, financial or material support from publishers (The following conflicts were disclosed)

None

8. Medical/Orthopaedic publications editorial/governing board (The following conflicts were disclosed)

None

9. Board member/committee appointments for a society (The following conflicts were disclosed)

None

**Each author must sign AND print or type his/her name, date and submit a separate form**

In addition, one BLINDED Conflict of Interest form (no author names used) should be submitted per manuscript with all author disclosures.


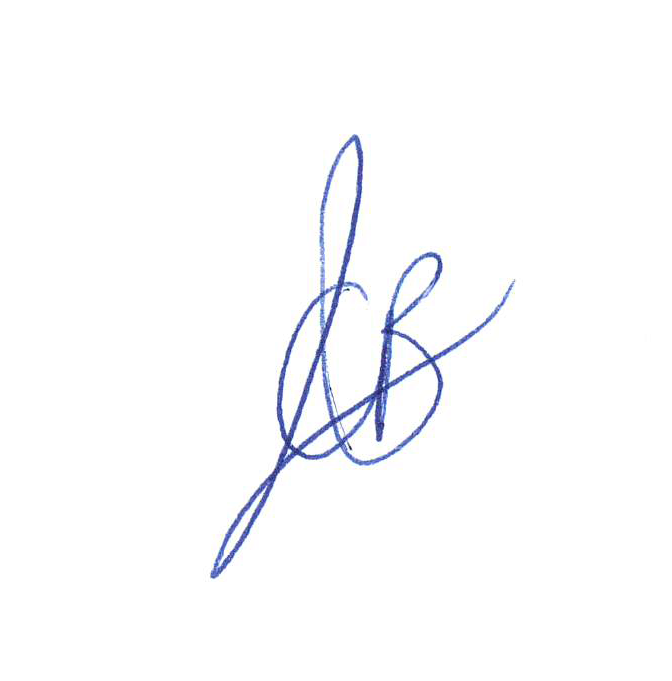


Christel Braaksma Author Signature Date: 25-8-2025
