# Supplementary material for: A Comparison of the Psychometric Properties of PROMIS Computer Adaptive Tests and Short Forms Vs Legacy Patient-Reported Outcome Measures in Total Knee Arthroplasty Patients
Source: Arthroplast Today. 2026 Mar 6;38:101964. doi: 10.1016/j.artd.2026.101964 (PMC12993152; doi:10.1016/j.artd.2026.101964)
Supplement: Conflict of Interest Statement for Terwee [file mmc2.docx]

# INDIVIDUAL CONFLICT OF INTEREST STATEMENT

***American Association of Hip and Knee Surgeons***

(Adopted from the American Academy of Orthopaedic Surgeons disclosure statement)

The following form **must be filled out completely and submitted by each author (example, 6 authors, 6 forms).**

**All items require a response. If there is no relevant disclosure for a given item, enter "*None*.”**

**Manuscript Title**

**A comparison of the psychometric properties of PROMIS computer adaptive tests and short forms versus legacy patient-reported outcome measures in total knee arthroplasty patients**

1. Royalties from a company or supplier (The following conflicts were disclosed) None

2. Speakers bureau/paid presentations for a company or supplier (The following conflicts were disclosed) None

3A. Paid employee for a company or supplier (The following conflicts were disclosed) None

3B. Paid consultant for a company or supplier (The following conflicts were disclosed) None

3C. Unpaid consultants for a company or supplier (The following conflicts were disclosed) None

4. Stock or stock options in a company or supplier (The following conflicts were disclosed) None

5. Research support from a company or supplier as a Principal Investigator (The following conflicts were disclosed) None

6. Other financial or material support from a company or supplier (The following conflicts were disclosed) None

7. Royalties, financial or material support from publishers (The following conflicts were disclosed) CB Terwee receives royalties from the text book Measurement in Medicine

8. Medical/Orthopaedic publications editorial/governing board (The following conflicts were disclosed) None

9. Board member/committee appointments for a society (The following conflicts were disclosed) CB Terwee is past president of the PROMIS Health Organization and head of the Dutch-Flemish PROMIS National Center

**Each author must sign AND print or type his/her name, date and submit a separate form**

In addition, one BLINDED Conflict of Interest form (no author names used) should be submitted per manuscript with all author disclosures.

CB Terwee
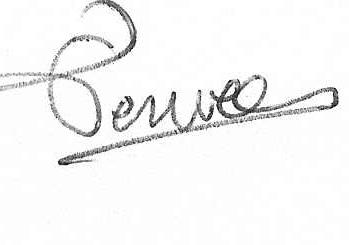
 18-8-2025

Author Name (Print or Type) Author Signature Date
